# *European Journal of Psychotraumatology* (EJPT): three years as an Open Access journal

**DOI:** 10.3402/ejpt.v4i0.23512

**Published:** 2013-12-20

**Authors:** Miranda Olff

**Affiliations:** Department of Psychiatry, Academic Medical Centre, University of Amsterdam & Arq Psychotrauma Expert Group, Diemen, the Netherlands

## Three years since launch

**Fig. 1:**
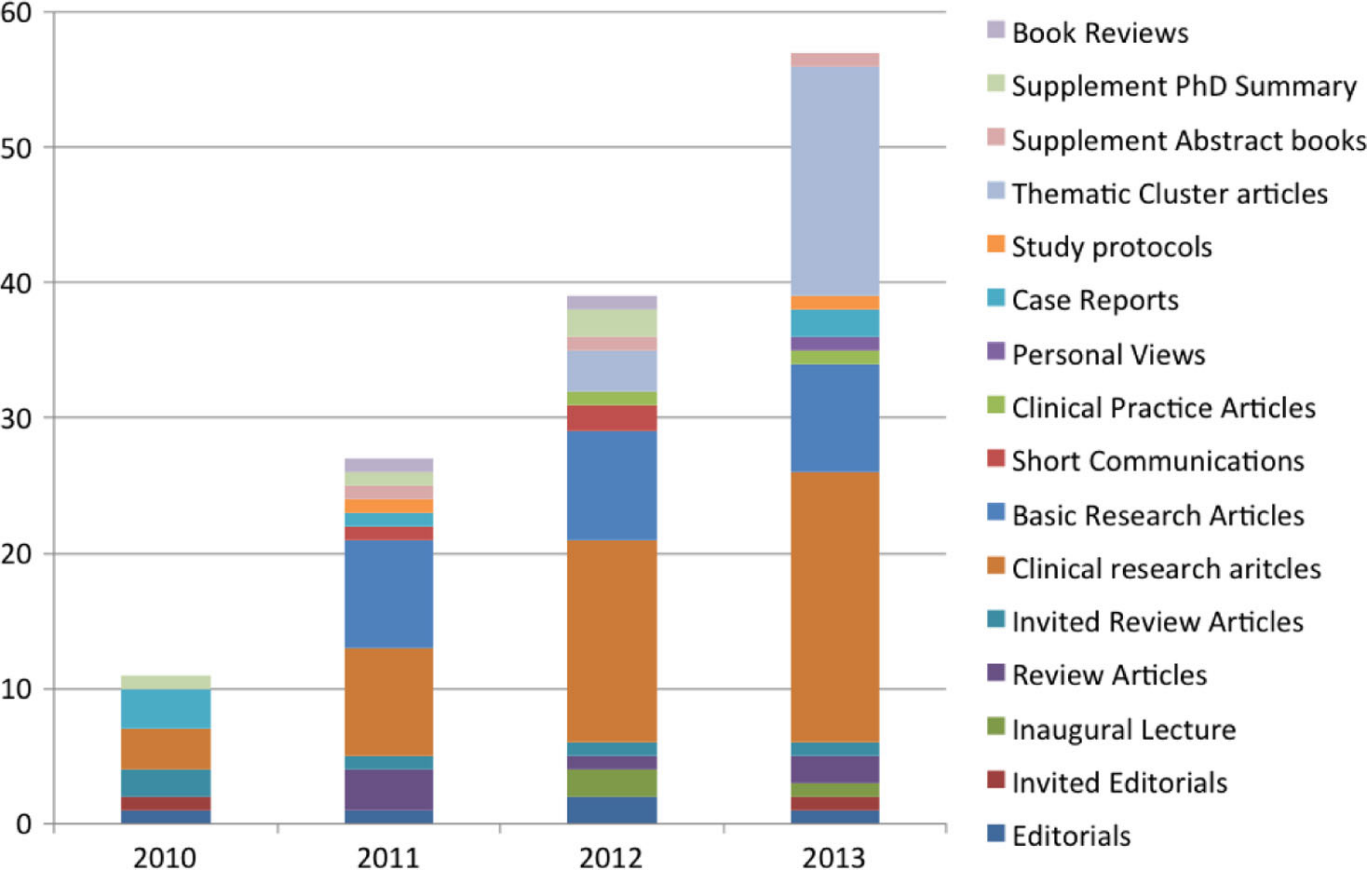
Number and types of published articles.

It is now three years since we launched the *European Journal of Psychotraumatology* (EJPT). In December 2010, a handful of articles were published, forming volume 1. Since then, *EJPT* has shown a strong increase in the number of publications, citations, and downloads (see Olff & Bindslev, 2011; Olff 2012). As of December 2013, a total of 134 articles have been published, including clinical and basic research articles, review articles, editorials, and thematic clusters, including supplements (Fig. 1).

The rejection rate is approximately 40%. We aim to have the initial review completed within 4 weeks. Accepted manuscripts are published in their final version online approximately 4 weeks following the final editorial decision, depending on how quickly authors respond to inquiries.

## What topics does *EJPT* address?

**Fig. 2:**
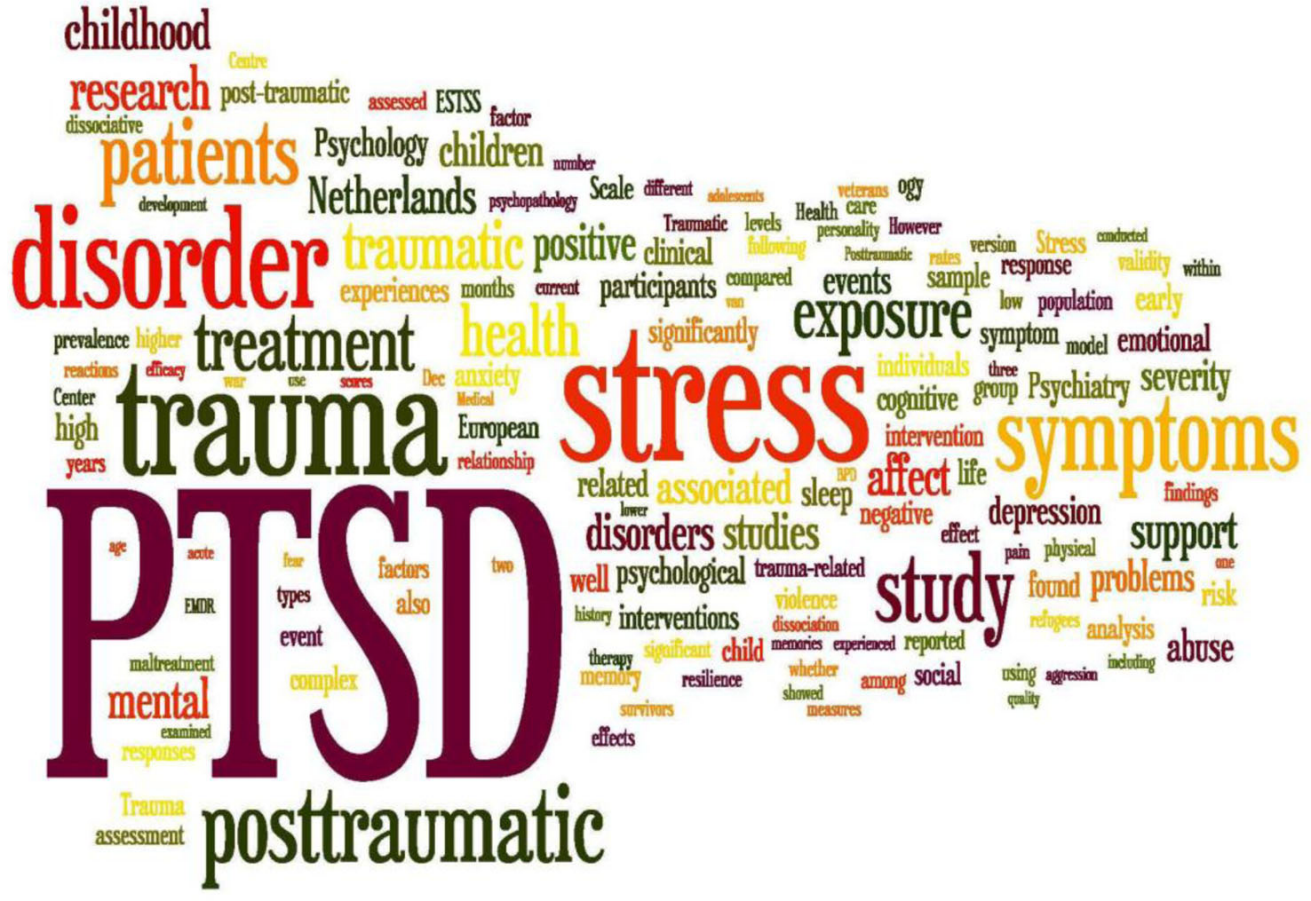
Topic cloud based on abstracts of articles published in EJPT since its launch in December 2010, created in Wordle™ (www.wordle.net).

A Wordle™ plot based on all abstracts published in *EJPT* to date (Fig. 2) shows that the main topics covered are, not surprisingly, PTSD, trauma, and traumatic stress. These conditions are discussed in both clinical and more basic research-oriented papers, and in both adults and children, in various settings. While some of the journal's best cited papers discuss brain circuits underlying (complex) PTSD (e.g., Frewen, Dozois, & Lanius, 2012; Lanius, Frewen, Vermetten, & Yehuda, 2010; Sack, Cillien, & Hopper, 2012; Novakovic et al., 2011), the plot shows that neurobiology is currently underrepresented and we would welcome a greater number of submissions in this subject area.

## Reaching potential audiences globally

Since launch, the *EJPT* website has been visited almost 80,000 times by a little more than 50,000 unique visitors representing 178 countries. The journal receives an average of approximately 6,000 full-text views of articles per month from the journal website in addition to a nearly equivalent number via PubMed Central. Those accessing the journal are most commonly from the USA, Netherlands, UK, Germany, Sweden, Canada, Australia, Norway, India, and Italy, closely followed by visitors from Switzerland, France, Denmark, Austria, Turkey, Poland, China, Israel, and Belgium.

To increase accessibility, the abstract of each article published in *EJPT* is translated into several languages, including French, Turkish, Spanish, German, Polish, and Russian. Upon request by our Chinese colleagues, we have now introduced Chinese translations of abstracts. Many thanks to our partners in Hangzhou, Drs Zhang, Liu, and Tan.

**Fig. 3:**
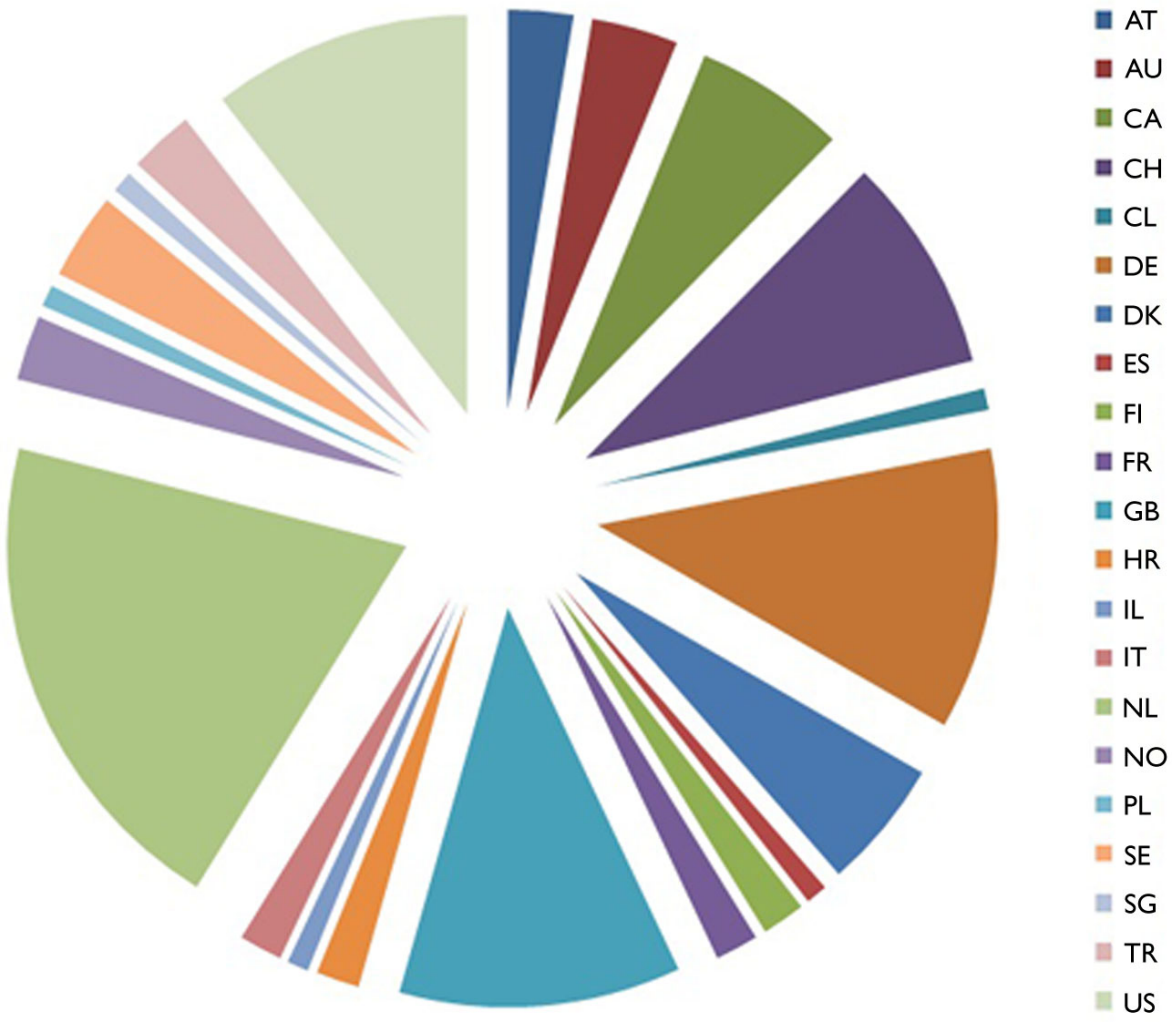
Global distribution of authors 2010–2013.

The current geographic distribution of *EJPT* authors reflects how the journal is accessed globally (see Olff & Vermetten, 2013). Figure 3 shows the current global distribution of authors over the past three years. It shows a wide distribution around the globe, but clearly we would hope that *EJPT* should serve areas better in particular where there is less access to subscription journals as in low income countries. The high number of articles from the Netherlands does reflect the interest in psychotraumatology in the Netherlands (see also the cluster of papers on psychotraumatology in the Netherlands; Vermetten & Olff, 2013) and possibly the Open Access friendly climate in the country. Although *EJPT* was free to publish with over the first 3 years of operation, low-income countries are underrepresented also in submission of articles to the journal. An article processing charge has now been introduced, but the journal budget includes a waiver fund such that we can continue to encourage authors from nations where the fee would be a burden.

## Has Open Access benefited the Journal?

We wished to establish a journal with a publication model that disseminates scientific knowledge about traumatic stress to as many researchers and other readers as possible around the world. Articles in *EJPT* are freely available to about two billion people worldwide via the journal's website and PubMed Central, without any need for registration or subscription.

In Europe, there is still a great need for such a journal, let alone the rest of the world. In October, I gave a lecture on Open Access publishing in the Ukraine. The discussions I experienced there made it absolutely clear that Eastern European countries have very limited access to scientific research and that they welcome free access. But also in other areas of the world, such as Africa or Asia, there is a need for free access to scientific results from psychotraumatology research. In short, it would be almost unethical *not* to make knowledge and data on psychotraumatology freely available. This raises the question of how we can finance open access to research literature in a sustainable way.

## A sustainable publication financial model?

*EJPT* is owned by the ESTSS. Unlike the case for some societies, the journal does not generate income for the society. On the contrary, ESTSS has invested heavily in *EJPT* to ensure a successful publishing outlet. There are costs associated with the production and online publication of all articles (e.g., copy-editing, typesetting, indexing, etc.), in addition to costs for promoting the journal, website maintenance, etc., work the publisher needs to do in order for *EJPT* to be a top scientific journal.

To cover these costs, *EJPT* takes advantage of a few different sources of income. Low APCs (article processing charges) have been introduced, which are below those typically charged by other open access publications. Members of ESTSS receive up to a 75% rebate as a member benefit. As mentioned above, ESTSS has financially supported the journal from the start of December 2010, in part, through a 3-year grant (2012–2014) from The Netherland's Organisation for Scientific Research (NWO) (Incentive Open Access fund). This initial support meant that we were able to waive fees for ESTSS members, which was helpful during the start-up phase.

It will soon be possible to make a donation to the ESTSS to support *EJPT* via a link from the journal's website. ESTSS makes it possible for the Society to receive such contributions as tax-exempt income.

## Mandatory Open Access in the future?

A growing number of funding agencies and research councils are requiring Open Access to publications based on the research they have supported, *and* they now provide funding to cover open access charges; see, for example, the Wellcome Trust policy or that of the British research councils, RCUK. Of special interest to European researchers is that the European Commission recently announced that Open Access will be mandatory for all scientific publications related to projects funded under Horizon 2020, the EU's Research & Innovation funding program for 2014–2020. Again, Open Access seems to be the way forward.

## What about copyright?

Hand in hand with the research funders’ demands for Open Access, many also require that all articles be published under the Creative Commons Attribution 3.0 Unported (CC BY 3.0) License, which means that anybody may copy and redistribute the material in any medium or format, and adapt it—remix, transform, and build upon the material—for any purpose, even commercially. As from 2014, *EJPT* will move from the Creative Commons Attribution-Noncommercial 3.0 Unported License to the CC-BY license.

## Indexing and impact factor

In academia, one thing tends to count above all else: the number of publications one has published in the highest ranking journals—highest ranking, that is, according to the Impact Factor. The impact factor still seems to determine the prestige of a journal, and by proxy, the work of individual authors who publish in that journal even though their article has never been cited. *EJPT* has applied for an impact factor, and the publisher's contact point at the Science Citation Database has indicated that we should have a decision by year's end. We are optimistic about receiving a response. We are aware that this is important to our publishing authors and will continue to press for a decision.

While we await an official impact factor, we are able to turn to alternative metrics. With the possibilities afforded by the Internet and tagging of online publications, other types of metrics are entering the scene that may well in the longer perspective outmanoeuvre the Impact Factor. *EJPT* has just recently introduced so-called Article-Level Metrics (ALM), based on a code developed by PLOS. After the abstract of each article, the author—and indeed anybody who is interested—can follow the number of downloads of his/her article, where and how many times it has been cited and referenced, if it has been shared through social media, and the number of comments it has received in various forums such as LinkedIn or Twitter. This is a very powerful tool for authors to judge the reach and influence of an article and perhaps share this information with peers, employers, and funders.

## Highlights of 2013

With almost 60 papers, it was not easy to choose, but the paper that was most mentioned in a voting round among *EJPT* and ESTSS board members is an article by Marylène Cloitre, Donn W. Garvert, Chris R. Brewin, Richard A. Bryant, Andreas Maercker (2013), entitled “Evidence for proposed ICD-11 PTSD and complex PTSD: a latent profile analysis”. The authors re-examined datasets of treatment-seeking patients and used a latent profile analysis (LPA) to determine whether there are classes of individuals that are distinguishable according to PTSD and complex PTSD symptom profiles. This is a timely paper that contributes to the discussion on how to best diagnose PTSD considering that the WHO ICD-11 (International Classification of Diseases, 11th revision) has proposed—unlike the DSM-5—that there should be two related diagnoses, PTSD and complex PTSD, within the spectrum of trauma and stress-related disorders. The authors do seem to confirm the ISC-11 proposal. The last paper published in December also addresses the proposed ICD-11 criteria for PTSD and complex PTSD and compared their prevalence to the ICD-10 PTSD prevalence (Knefel & Lueger-Schuester, 2013)

Other papers that stood out this past year are “Susceptibility to long-term misinformation effect outside of the laboratory” by Lommen, Engelhard, & van den Hout, (2013) which has already been accessed nearly 4,000 times, and “Trauma histories among justice-involved youth: findings from the National Child Traumatic Stress Network” by Dierkhising, Ko, Woods-Jaeger, Briggs, Lee, & Pynoos (2013), which has also been heavily downloaded and created some discussion on the Psychotraumatology LinkedIn Group.

## New in *EJPT*

We have introduced “Personal views”, a new section, for critical and thought-provoking contributions. The first one is an interview with Stevan Hobfall (Te Brake & Dückers, 2013), who holds views that certainly raise important questions.

Since the beginning of 2013, we are very pleased to have Dr Jane Herlihy, clinical psychologist and director of the Centre for the Study of Emotion and Law, London, UK, joining us as Associate Editor. She will share this job with Dr Stuart Turner. Also, we have a new statistics editor, Dr Rens van der Schoot, an expert in multivariate statistics, structural equation modeling, and Bayesian statistics. Please find details of the full editorial team here http://www.ejpt.net/index.php/ejpt/about/editorialTeam.

*Miranda Olff* Department of Psychiatry, Academic Medical Centre
University of Amsterdam & Arq Psychotrauma Expert Group Diemen, the Netherlands

## References

[CIT0001] Cloitre M, Garvert D, Brewin C, Bryant R, Maercker A (2013). Evidence for proposed ICD-11 PTSD and complex PTSD: A latent profile analysis. European Journal of Psychotraumatology.

[CIT0002] Dierkhising C, Ko S, Woods-Jaeger B, Briggs E, Lee R, Pynoos R (2013). Trauma histories among justice-involved youth: findings from the National Child Traumatic Stress Network. European Journal of Psychotraumatology.

[CIT0003] Frewen P, Dozois D, Lanius R (2012). Assessment of anhedonia in psychological trauma: psychometric and neuroimaging perspectives. European Journal of Psychotraumatology.

[CIT0004] Knefel M, Lueger-Schuester B (2013). An evaluation of ICD-11 PTSD and complex PTSD criteria in a sample of adult survivors of institutional abuse. European Journal of Psychotraumatology.

[CIT0005] Lanius R, Frewen P, Vermetten E, Yehuda R (2010). Fear conditioning and early life vulnerabilities: two distinct pathways of emotional dysregulation and brain dysfunction in PTSD. European Journal of Psychotraumatology.

[CIT0006] Lommen M, Engelhard I, van den Hout M (2013). Susceptibility to long-term misinformation effect outside of the laboratory. European Journal of Psychotraumatology.

[CIT0007] Novakovic V, Sher L, Lapidus K, Mindes J, Golier J, Yehuda R (2011). Brain stimulation in posttraumatic stress disorder. European Journal of Psychotraumatology.

[CIT0008] Olff M (2010). European Journal of Psychotraumatology: The European Society for Traumatic Stress Studies launches new journal. European Journal of Psychotraumatology.

[CIT0009] Olff M (2012). Advances in European Psychotraumatology. European Journal of Psychotraumatology.

[CIT0010] Olff M, Bindslev A (2011). One year later.…. European Journal of Psychotraumatology.

[CIT0011] Olff M, Vermetten E (2013). Psychotrauma research in the Netherlands. European Journal of Psychotraumatology.

[CIT0012] Sack M, Cillien M, Hopper J (2012). Acute dissociation and cardiac reactivity to script-driven imagery in trauma-related disorders. European Journal of Psychotraumatology.

[CIT0013] Te Brake H, Dückers (2013). Early psychosocial interventions after disasters, terrorism and other shocking events: Is there a gap between norms and practice in Europe?. European Journal of Psychotraumatology.

[CIT0014] Vermetten E, Olff M (2013). Psychotraumatology in the Netherlands. European Journal of Psychotraumatology.

